# Assessment of erythrocyte morphology in patients with type 2 diabetes mellitus: a pilot study of electron microscopy-based analysis in relation to healthy controls

**DOI:** 10.3906/sag-2103-336

**Published:** 2021-10-21

**Authors:** Tülay MORTAŞ, Şenay ARIKAN DURMAZ, Şaban Cem SEZEN, Yasemin SAVRANLAR

**Affiliations:** 1 Department of Histology and Embryology, Faculty of Medicine, Kırıkkale University, Kırıkkale Turkey; 2 Department of Endocrinology and Metabolism, Faculty of Medicine, Kırıkkale University, Kırıkkale Turkey; 3 Department of Histology and Embryology, Faculty of Medicine, Kırıkkale University, Kırıkkale Turkey; 4 Department of Histology and Embryology, Faculty of Dentistry, Nuh Naci Yazgan University, Kayseri Turkey

**Keywords:** Diabetes mellitus, erythrocyte, scanning electron microscopy, morphology, eryptosis

## Abstract

**Background/aim:**

The present study aimed to assess erythrocyte morphology in newly diagnosed type 2 diabetes mellitus patients using scanning electron microscopy.

**Materials and methods:**

In total, 30 patients admitted to endocrine outpatient clinics were included in the study. The patients were divided into two groups according to their fasting blood glucose levels: type 2 diabetes mellitus (n = 15, fasting blood glucose levels ≥ 126 mg/dL) and control (n = 15, fasting blood glucose levels < 99 mg/dL). The patient’s demographic characteristics, haemoglobin A1c levels, and scanning electron microscopy findings regarding erythrocyte morphology were recorded.

**Results:**

There was no significant difference between the control and type 2 diabetes mellitus group in terms of the participants’ age (51.13 ± 8.53 vs. 50.33 ± 8.72 years, p = 0.8) and the male/female ratio (9/6 vs. 9/6). In the control group, discocytes were abundant, echinocytes were rare, and spherocytes were absent. On the other hand, discocytes were less common and echinocyte-shaped erythrocytes were more common in the type 2 diabetes mellitus group than in the control group. In addition, spherocytes were detected in the type 2 diabetes mellitus group. Moreover, the diameter of discocytes was significantly lower (p = 0.014), and blood glucose and haemoglobin A1c levels were significantly higher (p < 0.05 for both) in the type 2 diabetes mellitus group than in the control group.

**Conclusion:**

Our findings indicate that high glucose levels in type 2 diabetes mellitus patients lead to significant alterations in erythrocyte morphology, including decreased erythrocyte deformability and the formation of echinocytes and spherocytes due to eryptosis. The possibility of decreased erythrocyte deformability due to excessive eryptosis may disturb microcirculation in newly diagnosed, treatment-naïve type 2 diabetes mellitus patients who do not have any complications**.**

## 1. Introduction

Globally, the prevalence of type 2 diabetes mellitus (T2DM) is high and continues to increase across all regions. This increase is driven by population ageing, economic development and increasing urbanization, which have resulted in more sedentary lifestyles and increased consumption of unhealthy foods linked to obesity [1]. In 2017, approximately 462 million individuals were affected by T2DM; this number corresponds to 6.28% of the world’s population or to a prevalence rate of 6059 cases per 100,000 population [2]. In T2DM patients, the haematological system is vulnerable to oxidative stress because of characteristically high glucose levels, which can cause haemoglobin molecules localized in erythrocytes to be nonenzymatically glycosylated, thereby affecting erythrocyte morphology and functions [3,4].

The biconcave disc shapes of erythrocytes provide a greater surface area/volume ratio for the efficient diffusion of respiratory gases [5]. Moreover, erythrocyte deformability plays a critical role in determining blood viscosity, microvascular tissue perfusion, and oxygen delivery [5–8]. Erythrocyte deformability can be attributed to cytoskeletal structures on erythrocyte membranes [9]. Biconcave disc shapes and deformability are extremely important for the functioning of erythrocytes. Before ageing, erythrocytes may be exposed to factors that can negatively impact their integrity, function, and lifespan. In such situations, erythrocytes may undergo an apoptosis-like process known as eryptosis, a type of cell death, because of the lack of nuclear condensation and mitochondrial depolarization [10]. The increase in reactive oxygen species (ROS) is an important indicator of oxidative stress, which can accelerate eryptosis [11,12]. Erythrocytes are sensitive to oxidative damage because they contain iron and several unsaturated fatty acids [9]. During eryptosis, the cell loses its biconcave disc shape, shrinks and releases extracellular vesicles [13]. In this process, Ca^2+^ is released by the activation of permeable nonselective cation channels, following which the cytosolic Ca^2+^ concentration increases, cell membrane phospholipid asymmetry gets disrupted, and phosphatidylserine translocation occurs on the erythrocyte surface [14,15]. Eryptosis affects the biconcave disc shape and deformability of erythrocytes, resulting in smaller, fragile, and unstable erythrocytes. 

The present study was designed to comparatively evaluate eryptosis in newly diagnosed T2DM patients and healthy controls using scanning electron microscopy (SEM). SEM is a useful and specialized method to study changes in the physical structure of erythrocytes associated with eryptosis [11]. We assessed erythrocyte morphology on the basis of the classification provided by Bessis [16] using SEM. We evaluated eryptosis in both the groups according to the erythrocyte morphology observed in SEM images. 

## 2. Materials and methods

### 2.1. Study population

In total, 15 patients (nine males and six females) aged between 40 and 65 years who were newly diagnosed with T2DM upon admission to the Endocrinology Outpatient Clinic, Kırıkkale University Faculty of Medicine between June 2015 and June 2018 were included in the T2DM group.

The control group consisted of 15 age- and sex-matched healthy volunteers (nine males and six females). T2DM was diagnosed as per the American Diabetes Association criteria [17]. According to these criteria, T2DM is defined as glucose levels ≥ 126 mg/dL, haemoglobin A1c (HbA1c) levels ≥ 6.5%, 2 h postprandial glucose levels ≥ 200 mg/dL in an oral glucose tolerance test (75 g glucose) or random glucose levels ≥ 200 mg/dL in plasma samples collected between 8.00 and 10.00 a.m. from a person with diabetic symptoms after a minimum of 8 h of fasting. 

Nonsmoker patients who did not use any medication, including vitamins and herbal products, who were not pregnant; who did not have any other health problems, including high blood pressure, who did not have a history of blood transfusion or haematological disease, alcoholism, or malignancy, and who did not have any active infection were included in the study.

Written informed consent was obtained from each participant, and the participants’ names were coded to protect their identity. Approval to conduct the study was obtained from the local ethics committee (date of approval: 15.06.2015, protocol no: 16/04).

### 2.2. Collection and preparation of blood samples for SEM

Peripheral venous blood samples were collected into 2.5 cc citrated tubes from the brachial veins of the participants in a sitting position in the morning after at least 8 h of fasting. The tubes were immediately centrifuged for 5 min at 4000 rpm without delay. In order to obtain 500,000 erythrocytes per mm^3^ of blood, 500 µL of the centrifuged sample was slowly drawn from the bottom of the centrifuge tube (i.e. the part where erythrocytes settle) and transferred to another tube [18]. Following this, 500 µL of saline was added to the tube in order to ensure homogeneity. Next, 500 µL of the sample was drawn from this tube and further transferred to a new tube. For SEM assessment, the transferred blood samples were washed with phosphate buffer to remove excess proteins [11]. For this purpose, 500 µL of phosphate buffer was added to the samples, followed by centrifugation at 3000 rpm for 8 min. The top pellet of the centrifuged samples was removed using a pipette and discarded. The erythrocytes were washed with phosphate buffer thrice in total. Following this, a fixation solution consisting of 1000 µL gluteraldehyde and 17000 µL phosphate buffer was prepared. Next, 500 µL of this solution was added to the erythrocytes in the tubes and incubated for 2.5 h to fix the erythrocytes. One milliliter of the final suspension was air dried on a glass coverslip (1 cm diameter) glued to an aluminum scanning electron microscope stub.

### 2.3. Imaging of erythrocytes by SEM

Samples were coated with a thin layer of gold and examined at 20 kV using SEM (JEOL JSM-5600 MP17400041, JEOL, Japan) at Kırıkkale University of Scientific and Technological Research Center. The images of 15 patients (two different images per patient) from each group were evaluated using electron microscopy. For statistical analysis, random areas were selected from each sample and images were taken at 1500 × and 9000 × magnifications.

### 2.4. Evaluation of erythrocyte morphology by SEM

Erythrocyte morphology was examined using SEM, which provides a 10-fold higher resolution than light microscopy [7]. Images obtained using SEM were evaluated on the basis of the presence or absence of discocyte, echinocyte and spherocyte shapes [16,19,20]. The number of discocytes, echinocytes, and spherocytes was counted in the images. Erythrocyte shapes that were not properly visible in the images were not included in the count. In addition, the diameters of discocytes were measured in both groups. The data were then subjected to statistical analyses.

### 2.5. Statistical analysis

All statistical analyses were performed using the Statistical Package for the Social Sciences (SPSS) version 20 package for Windows (SPSS Inc., Chicago, IL, USA). The Mann–Whitney U test was used to compare continuous variables between the groups. Categorical variables were compared using chi-square and Fisher exact tests, where applicable. A p value < 0.05 was accepted as statistically significant.

## 3. Results 

Demographic characteristics and SEM findings of the control group are presented in Table 1, while those of the T2DM group are presented in Table 2.

**Table 1:** Demographic characteristics, glycaemic data, and erythrocyte morphology findings in the control group (n = 15).

	Control subject codes														
	SAB	SK	KÜ	AKA	KD	NG	MA	HFG	TZA	DBSK	OA	FP	BK	GA	MFÖ
Age (year)	40	42	57	43	55	42	48	53	58	44	63	42	59	65	56
Sex	Female	Male	Male	Male	Male	Male	Female	Female	Male	Female	Male	Male	Female	Female	Male
Discocyte diameter(µm)	7.006.005.776.557.006.407.186.637.176.67	6.406.546.846.607.456.486.226.995.845.31	6.607.417.076.495.997.207.256.916.537.31	5.506.696.235.475.956.406.576.036.478.14	7.256.706.137.607.477.047.075.878.116.67	6.276.136.796.535.857.547.356.206.395.57	6.986.406.286.186.097.517.156.176.856.53	6.296.545.606.456.677.477.436.836.385.86	5.745.826.796.276.136.416.446.266.985.65	6.74 6.876.406.627.876.866.286.806.966.93	5.495.995.846.376.036.466.327.135.735.67	7.367.276.406.546.326.286.807.317.635.78	6.086.927.387.928.457.477.957.558.066.60	6.326.276.806.517.626.296.796.807.417.04	6.676.936.377.387.167.667.617.075.735.27
Discocyte count	74	89	68	88	69	70	61	97	96	62	87	47	55	62	59
Echinocyte count	0	24	24	36	8	10	7	8	7	2	1	1	2	18	2
Spherocyte count	0	0	0	0	0	0	0	0	0	0	0	0	0	0	0
Spherocyte diameter (µm)	-	-	-	-	-	-	-	-	-	-	-	-	-	-	-
Glucose (mg/dL)	85.30	93.63	91.18	97.46	92.64	94.54	91.73	92.95	95.81	87.41	90.18	94.68	93.66	94.31	94.53
HbA1c (%)	5.15	5.27	5.11	5.17	5.13	5.27	5.19	5.15	5.03	5.10	4.97	5.11	5.3	5.2	5.3

**Table 2:** Demographic characteristics, glycaemic data, and erythrocyte morphology findings in the T2DM group (n = 15).

	T2DM Patient codes														
	KA	YV	İG	Hİ	İsG	MŞ	ST	NÇ	ZM	SÇ	FS	TÜ	ÖK	İV	MT
Age (year)	53	56	41	41	59	62	57	40	63	52	43	40	40	50	58
Gender	Female	Male	Male	Female	Male	Male	Female	Male	Female	Male	Female	Male	Male	Female	Male
Discocyte diameter (µm)	-	-	6.806.285.735.975.676.806.626.607.885.07	5.345.605.815.206.085.575.876.256.367.18	6.676.566.406.925.705.785.535.315.605.65	6.476.036.845.507.045.735.786.236.257.39	6.206.226.466.055.736.696.226.325.845.73	6.296.077.637.607.107.926.445.778.946.32	6.276.536.025.705.746.386.277.187.285.95	6.27 6.076.365.877.717.047.106.796.275.66	-	-	5.475.525.576.026.095.375.475.875.076.15	-	-
Discocyte count	0	0	10	11	11	10	13	11	49	10	0	0	119	0	0
Echinocyte count	162	48	125	66	105	98	93	96	15	86	130	207	1	136	118
Spherocyte count	17	164	0	0	0	0	0	0	0	0	125	43	0	76	10
Spherocyte diameter (µm)	4.534.534.484.534.134.614.014.084.583.77	3.763.603.87 3.47 3.50 3.59 3.73 3.64 3.47 3.58	-	-	-	-	-	-	-	-	3.943.943.873.643.694.954.154.154.224.37	3.523.593.333.333.333.593.543.473.723.52	-	3.743.853.814.184.064.064.334.963.593.73	4.704.734.004.124.794.254.534.484.704.54
Glucose (mg/dL)	254	142	255.42	126	199	262	126	370	131	159	137	226	280	166	175
HbA1c (%)	9.3	6.9	11.1	5.7	10.4	11	9.1	10.4	11.5	8.8	6.7	11.6	12.4	8.1	9.4

Note: The participants’ names were coded to prevent their identification (e.g., KA, YV and so on). HbA1c = Haemoglobin A1c, T2DM = Type 2 diabetes mellitus.

### 3.1. Demographic and glycemic data in study groups

There was no significant difference between the control and the T2DM group in terms of the participants’ age (51.13 ± 8.53 vs. 50.33 ± 8.72 years, p = 0.8) and the male/female ratio (9/6 vs. 9/6), respectively.

The fasting blood glucose levels in the T2DM group (range, 126–370 mg/dL) were significantly higher than those in the control group (range, 85.30–97.46 mg/dL) (Mann–Whitney U test, p < 0.05). Similarly, the HbA1c levels in the T2DM group (range, 5.7%–12.4%) were significantly higher than those in the control group (range, 4.97%–5.3%) (Mann–Whitney U test, p < 0.001).

### 3.2. SEM images in the control group

Discocytes and echinocytes were detected in the control group. However, spherocytes were not detected in this group. As discocytes have a stable membrane structure, their diameters were measured. 

**Figure 1:**
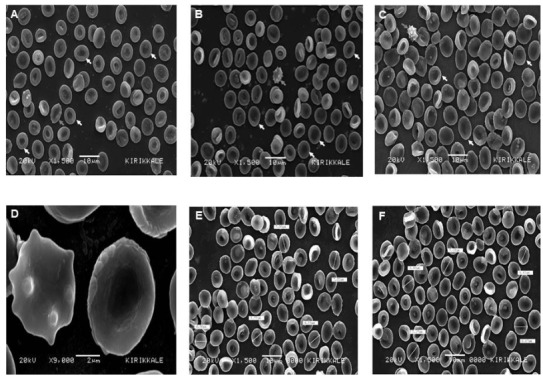
Erythrocyte morphology in the control group, as assessed using scanning electron microscopy. A) TZA-coded control participant with commonly detected discocytes (white arrows) and echinocytes (grey arrows). B) FP-coded control participant with discocytes (white arrows) and echinocytes (grey arrows). C) NG-coded control participant with spiny echinocytes (grey arrows) and discocytes (white arrows). D) KU-coded control participant with echinocytes (left) and discocytes (right). E) SAB-coded control participant in whom the diameters of discocytes were measured. F) SAB-coded control participant in whom the diameters of discocytes were measured in a different area. Note: TZA, FP, NG, KU, SAB are the coded patient names.

Discocytes were the most common erythrocytes in all participants in the control group (Figure 1A). The cell membrane at the surface of discocytes was quite smooth. The middle region of discocytes was darker than its surroundings (Figures 1B–1D). The diameters of 10 randomly selected discocytes were measured for each participant; the diameters were found to range from 5.27 to 8.45 µm (Table 1, Figure 1E and 1F). In addition to discocytes, echinocytes were detected in the control group. While no echinocytes were detected in one participant, one to two echinocytes were detected in five participants, and seven to eight echinocytes were detected in four participants in the control group. Moreover, 24 echinocytes were detected in two participants and 10, 18, and 36 echinocytes were detected in the remaining three participants in the control group (Table 1). In other words, the number of echinocytes was higher in some participants in the control group. The morphological forms of echinocytes also varied (Figures 1A–1C), with membrane fluctuations being noted in the early stages and a spiny membrane structure being noted in the later stages (Figures 1B and 1C). The spiny echinocytes appeared to be slightly shrunken (Figure 1C). The center of discocytes appears dark, while the surrounding area appears light. There was no such color change in echinocytes (Figure 1D). The diameters of echinocytes could not be measured because they did not have a fixed membrane structure. As none of the participants in the control group had erythrocytes in the form of spherocytes, data on the diameters of spherocytes could not be recorded for the control group.

### 3.3. SEM images in the T2DM group

Discocytes, echinocytes, and spherocytes were detected in the T2DM group. Echinocytes were the most common erythrocytes in the T2DM group. Moreover, the number of spherocytes in the T2DM group was considerably higher than that in the control group (Table 2). As discocytes and spherocytes had a stable membrane structure, their diameters were measured.

**Figure 2:**
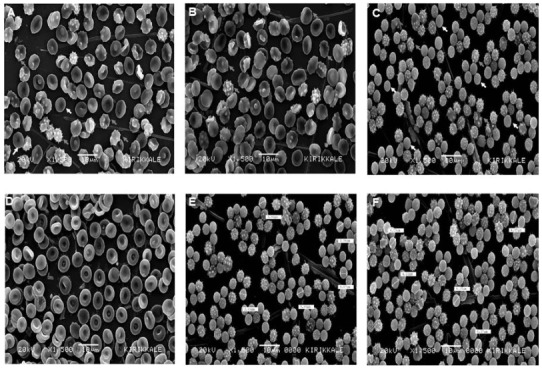
Erythrocyte morphology in the T2DM group, as assessed using scanning electron microscopy. A) IsG-coded T2DM patient with decreased discocytes (white arrows) and increased echinocytes (grey arrows). B) ST-coded T2DM patient with increased echinocytes (grey arrows). C) FS-coded T2DM patient with only echinocytes (grey arrows) and spherocytes (white arrows). D) ÖK-coded T2DM patient with plenty of discocytes. E) FS-coded T2DM patient in whom the diameters of spherocytes were measured. F) FS-coded T2DM patient in whom the diameters of spherocytes were measured in a different area. Note: IsG, ST, FS, ÖK are the coded patient names.

The number of discocytes in the T2DM group was lower than that in the control group (Figures 2A and 2B). On the other hand, there was an increase in the number of echinocytes and spherocytes in the T2DM group (Figures 2A–2D). While no discocytes were detected in six patients in the T2DM group, 10 discocytes were detected in three patients, 11 in three patients, 13 in one patient, 49 in one patient, and 119 in one patient in this group (Table 2). The diameters of discocytes ranged from 5.07 to 8.94 µm. According to the Mann–Whitney U test, the diameter of discocytes was significantly lower in the T2DM group than in the control group (p = 0.014). The number of echinocytes was higher in the T2DM group than in the control group. However, interestingly, 119 discocytes and one echinocyte were detected in one patient in the T2DM group (Table 2). The erythrocyte electron microscopy image for this patient in the type 2 diabetes mellitus group was similar to the control group. The diameters of echinocytes were not measured in the T2DM group. While the number of spherocytes was quite high in two patients, no spherocytes were detected in nine patients in the T2DM group (Table 2). The diameters of spherocytes with a fixed membrane structure were measured in this group. However, as spherocytes were not detectedin the control group, the diameters of spherocytes could not be compared between the two groups. The diameters of spherocytes ranged from 3.33 to 4.96 µm in the T2DM group (Figure 2E and 2F). Given the decrease in the number of discocytes and the very small diameters of spherocytes, the reduction in the diameters of erythrocytes seems to be notable in the T2DM group. 

## 4. Discussion 

In the present study, we found that discocytes were more common in the control group, while echinocytes were more common in the T2DM group. In addition, spherocytes were detected in the T2DM group. As a result of the shrinkage of echinocytes and their transformation into spherocytes in the T2DM group, there was a prominent reduction in their diameters. The decrease in diameters indicated that erythrocytes underwent extracellular vesiculation due to membrane loss. These morphological markers indicate the presence of eryptosis and resultant erythrocyte destruction in the T2DM group. If eryptosis is not balanced by erythropoiesis, it can cause anaemia. Erythropoiesis is a dynamic process, with 10 erythrocytes being estimated to be produced and removed from the circulation each day [10,13]. As long as eryptosis is compensated by a similar rate of erythropoiesis, the number of erythrocytes in the circulation will remain unchanged. In the present study, we did not observe morphological changes, such as a decrease in discocytes, an increase in echinocytes and the presence of spherocytes, in one of the patients in the T2DM group, although very high fasting blood glucose (280 mg/dL) and HbA1c (12.4%) levels were detected in that patient. Surprisingly, erythrocyte morphology was not affected by the high glucose and HbA1c levels in that patient. Considering the significant variation in the half-life and deterioration rate of erythrocytes between individuals [21], the possibility of differences in the eryptosis mechanism also seems likely.

The effects of systemic and autoimmune diseases and medications on eryptosis have been assessed in several studies. For instance, Egler et al. [22] used perifosine, a drug that triggers apoptosis, and investigated whether perifosine can trigger eryptosis. They concluded that perifosine can trigger eryptosis because of the activation of kinases with the entry of Ca^2+^. In addition, Bartolmas et al. [23] reported that eryptosis can occur in patients with cold autoimmune haemolytic anaemia and warm autoimmune haemolytic anaemia and that it frequently occurs in the presence of autoantibodies. They suggested that eryptosis can cause chronic haemolysis and anaemia and that reasonable treatment can be provided using erythropoietin. Furthermore, Saradamma et al. [9] examined the effect of alcohol on erythrocyte morphology and detected a change in the morphology of erythrocytes to stomatocytes and echinocytes as a result of lipid peroxides, protein carbonyls and altered membrane lipid and protein compositions. Moreover, Gyawali et al. [19] assessed the changes in erythrocyte morphology in patients with metabolic syndrome using SEM. They found a correlation between erythrocyte morphology and oxidative stress and inflammation in these patients. They also reported that patients with metabolic syndrome exhibit abnormal erythrocyte morphology and stated that changes in erythrocyte morphology due to oxidative stress and chronic inflammation can decrease the blood flow rate in capillaries with erythrocyte fragility.

The present study is the first to provide data on newly diagnosed, treatment-naïve T2DM patients, unlike previous studies on diabetic patients with advanced diabetic complications. Brown et al. [24] found early impaired erythrocyte deformability in T2DM patients with normal renal function. They also compared the renal function of nondiabetic and diabetic participants with kidney failure and found that T2DM patients with kidney failure had more impaired erythrocyte deformability. In addition, Nayak et al. [18] identified an association between the erythrocyte membrane lipid composition and serum lipid composition in T2DM patients with and without nephropathy and concluded that diet and lifestyle play important roles in lipid changes in the serum and erythrocyte membranes. Cahn et al. [25] assessed whether diabetic foot is associated with impaired erythrocyte deformability; they used cell flow cytometry to assess the distribution of deformation in erythrocytes and concluded that diabetic foot is associated with a decrease in erythrocyte deformability. Furthermore, Prestes et al. [26] stated that methylglyoxal, which plays an important role in the pathogenesis of T2DM, causes fragility and haemolysis in the erythrocyte membrane. Moreover, Moutzouri et al. [27] reported that impaired erythrocyte deformability could be further affected in septic T2DM patients and revealed the causative role of microcirculation in functional disorders. 

In the present study, we considered the possibility that oxidative stress caused by high glucose levels led to eryptosis in the T2DM group. Erythrocyte deformability decreased with the formation of echinocytes and spherocytes as a result of eryptosis. With an increase in the cytosolic Ca^2+^ concentration due to oxidative stress, phosphatidylserine translocation occurred on the erythrocyte surface because of the disruption of cell membrane phospholipid asymmetry as a result of the activation of K^+^ sensitive to Ca^2+^ [28]. Eventually, the erythrocytes in the T2DM group reduced in size and more fragile, small spherocytes appeared. Spherocyte formation occurred as a result of extracellular vesiculation. This process is irreversible because of the loss of cell membrane due to vesiculation. 

In the present study, we did not classify the extracellular vesicles. Supporting the externalisation of phosphatidylserine during the initiation of eryptosis using annexinV would have further strengthened our data. The presence of a small number of echinocytes in the control group confirmed that approximately 0.5% of circulating erythrocytes expose phosphatidylserine at their surface [23]. In fact, erythrocytes also undergo eryptosis in the normal state. Clinically, excessive eryptosis has been associated with the pathogenesis of anaemia, impaired microcirculation, or prothrombotic risk associated with a wide range of human diseases [10]. 

The present study has some limitations. First, this was a pilot, limited-population study. Second, the extent of cigarette consumption was self-reported; no biomarker was used to verify tobacco exposure. We could not include people who used drugs for various reasons. 

In conclusion, our findings in newly diagnosed, treatment-naïve T2DM patients with no diabetes-related complications indicate the presence of increased eryptosis and the resultant loss of erythrocyte deformability and impaired microcirculation. Therefore, the oxygen retention capacity of erythrocytes may also be decreased in such patients.

## Informed consent

Study protocol was approved by Kırıkkale University Local Ethics Committee (date of approval: 15.06.2015, protocol no: 16/04), and the study was performed according to the Declaration of Helsinki.
